# Giving what they want: How congruence between expected feedback quality and delivered feedback quality influences leader-member exchange and job performance

**DOI:** 10.3389/fpsyg.2023.1115861

**Published:** 2023-02-15

**Authors:** Qi Qi, Yanyan Liu, Zhaoyan Liu

**Affiliations:** ^1^Digital Management and Behavioral Decision Art Laboratory, Shandong University of Finance and Economics, Jinan, China; ^2^Business School, Nankai University, Tianjin, China

**Keywords:** feedback quality, leader-member exchange, task performance, OCB, learning goal orientation 2/47

## Abstract

As an attempt to solve the mixed results between leader feedback quality and employee job performance, this study proposes that employees’ expected feedback quality plays a key role in how employees react to leader feedback. Specifically, drawing on needs-supplies fit and social exchange theory, we posit that congruence between expected feedback quality and delivered feedback quality positively relates to employee task performance and organizational citizenship behavior (OCB) through leader-member exchange (LMX). Further, we posit that learning goal orientation may strengthen the positive effect of congruence between expected feedback quality and delivered feedback quality on LMX. Multi-wave data collected from 226 employees from China showed that congruence between expected feedback quality and delivered feedback quality improves LMX and in turn benefits task performance and OCB. Moreover, learning goal orientation intensifies the indirect effect of congruence between expected feedback quality and delivered feedback quality on task performance and OCB through LMX. The theoretical and practical implications of these findings are discussed.

## Introduction

Nowadays work conditions become increasingly uncertain and dynamic due to economic downturns, and fierce global competition. To adapt to such uncertainty and dynamics successfully and perform well, employees go beyond formal performance appraisals to informal feedback exchanges with leaders to judge how well they are meeting their performance goals (Ashford et al., 2016; Dahling et al., 2017; Li et al., 2018; Lan et al., 2020). Some research posits that feedback from leaders is instrumental for employees to improve job performance because feedback conveys desired behaviors by the organization and contains an evaluation of the quality of relevant work behaviors (London, 2003; Steelman et al., 2004). However, previous research has found that not all feedback or seeking feedback from leaders could result in increased job performance (Balcazar et al., 1985; Kluger and DeNisi, 1996; Anseel et al., 2015; Evans and Dobrosielska, 2021). A recent meta-analysis also confirms that despite feedback is a robust intervention, feedback did not uniformly increase performance (Sleiman et al., 2020). As explained by Aguinis et al. (2012), when feedback provided by leaders is beyond employees’ potential to improve, feedback will fail to improve employee performance. Murphy (2020) also believed that adding more feedback to employees might do little more than increase their stress. Besides, some scholars argued that an effective feedback environment is likely to result in increased job performance (Steelman et al., 2004). However, previous research suggests mixed results between feedback quality, a dimension of the feedback environment, and employee job performance. For example, Guo and Ling (2020) found that feedback quality is positively related to organizational citizenship behavior (OCB) and has no significant relationship with task performance. However, in an experimental study, Drouvelis and Paiardini (2022) found that feedback quality has positive effects on employee task performance.

Some scholars suggested that the impact of leader feedback on job performance more critically depends on employees’ reaction to feedback than the feedback itself (Jawahar, 2006; Rasheed et al., 2015), but little attention has been given to consider whether feedback quality meets employees’ expectations, a specific type of reaction to the feedback (Maden et al., 2016). Thus, to deepen our knowledge of the relationship between feedback quality and job performance, this study proposes that employees’ expected feedback quality plays a key role in how employees react to leader feedback. Feedback quality refers to “the extent to which the feedback is relevant, consistent, specific, and detailed in terms of helping improve one’s job performance” (Steelman et al., 2004, p. 167). Referring to this definition, we define expected feedback quality as the degree to which employees expect feedback quality from the direct leader to help them improve their job performance, regardless of whether it is positive or negative. Drawing on the needs-supplies fit theory (Kristof, 1996), when leaders’ supplies meet employees’ needs, employees tend to exhibit positive work attitudes such as job satisfaction (Klaic et al., 2018) and organizational commitment (Cao and Hamori, 2020), and work behaviors such as task performance and OCB (Travaglianti et al., 2017). According to this theory, expected feedback quality represents a need, and delivered feedback quality by the direct leader can be positioned as a supply. In the case of giving feedback quality, when leaders’ delivered feedback quality is consistent with employees’ expectations, employees could get relevant, specific, and detailed information to make their job performance progress (Steelman et al., 2004) Thus, this study posits that congruence between expected feedback quality and delivered feedback quality would positively relate to job performance.

To get a deeper understanding of the relationship between congruence between expected feedback quality and delivered feedback quality and job performance, this study further explores why and when congruence between expected feedback quality and delivered feedback quality affects job performance. As high-quality leader-member exchange (LMX) results from leaders’ supplies fulfilling employees’ expectations and will motivate employees to perform better (Gouldner, 1960), this study thus posits that congruence between expected feedback quality and delivered feedback quality is positively related to LMX, which in turn, enhances job performance. Moreover, the needs-supplies fit theory suggests that the extent to which fit influences employees’ attitudes would be contingent on how much employees valued the supplies (Edwards and Shipp, 2007). Since learning goal orientation, a disposition to develop competence by acquiring new skills and mastering new situations (Dweck, 1986), affects how much employees value the feedback (Brett and Atwater, 2001), this study, therefore, posits that the effect of congruence between expected feedback quality and delivered feedback quality on job performance through LMX is contingent on employees’ learning goal orientation.

This study makes several contributions to the literature. First, unlike most studies that advocated that more feedback leads to better job performance (Drouvelis and Paiardini, 2022), we add to previous work that shows that more feedback is not always better for job performance (Azmat and Iriberri, 2016; Lam et al., 2017; Guo and Ling, 2020). Derived from the needs-supplies theory of the employee perspective, this study not only identifies a specific needs-supplies target (i.e., feedback quality) but also provides a more detailed approach to understanding how feedback quality influences job performance by adopting polynomial regression analyses and response surface graphs.

Second, to our knowledge, few scholars have directly examined how the leader’s fulfillment of employee feedback needs affects LMX quality (Marstand et al., 2017; Masterson and Lensges, 2015). However, based on social exchange theory (Gouldner, 1960), LMX quality is based on one party providing the other party with a service or something that the other party values (Wayne et al., 1997). Marstand et al. (2017) also demonstrate that complementarity (and mutuality) is at the heart of LMX theory showing that complementary fit (i.e., congruence between expected feedback quality and delivered feedback quality) is important for LMX quality. Thus, drawing from the LMX literature, we reveal that LMX serves as the mechanism linking congruence between expected feedback quality and delivered feedback quality to job performance, providing a relational lens to understand why congruence between expected feedback quality and delivered feedback quality affects employee outcomes.

**Figure 1:**
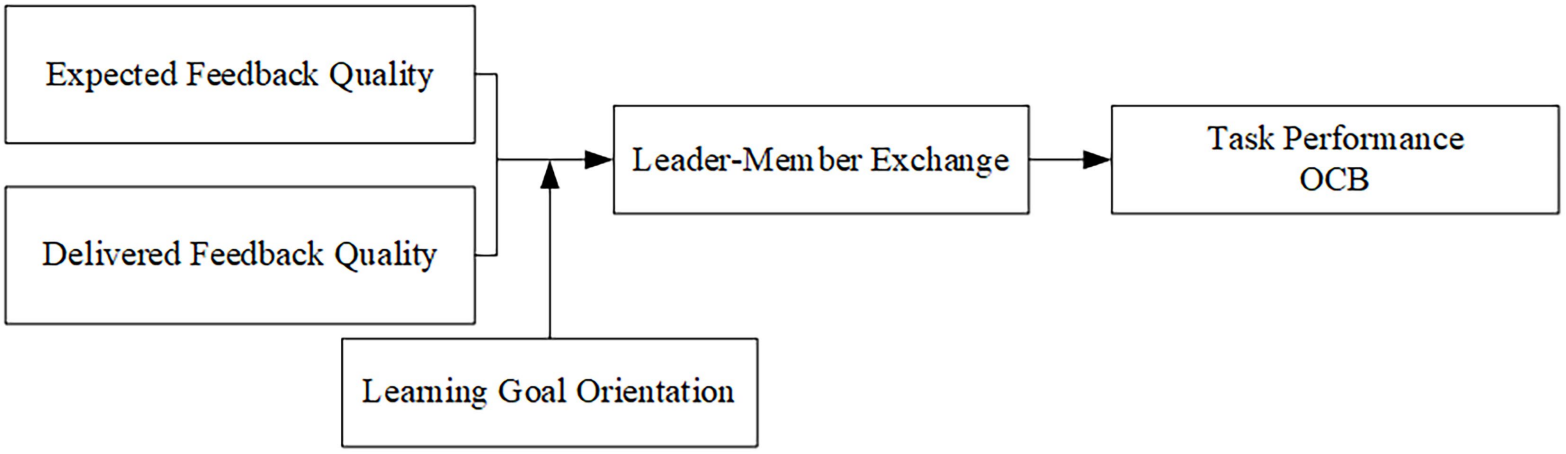
Hypothesized research model.

Third, following future suggestions of van Vianen (2018) in the person-environment fit filed to find what are the possible moderators of fit-performance, this study identifies employees’ learning goal orientation as a boundary condition in the relationship between congruence between expected feedback quality and delivered feedback quality and job performance. Such investigation not only helps recognize which personal attributes are relevant for fit perceptions (Van Vianen, 2018) but also provides a more nuanced picture of when congruence between expected feedback quality and delivered feedback quality influences job performance, allowing us to understand who is sensitive to the congruence between expected feedback quality and delivered feedback quality. The conceptual model of this study is shown in Figure 1.

## Theory and hypotheses

### Congruence between expected feedback quality and delivered feedback quality and LMX

As information delivered from leaders’ feedback is beneficial for employees to accurately view themselves, reach job performance standards, and improve social standing and relationships (Ashford et al., 2003; Lam et al., 2017), employees generally expect that leaders could provide valuable feedback (Ashford et al., 2016). However, research on feedback largely ignores the element of employees’ expectations in exploring the implications of leader feedback. As a critical domain of person-environment fit, needs-supplies fit shows the extent to which people’s needs are fulfilled by supplies in the environment. Specifically, when employees’ needs are congruent with those supplied by the organization, they will experience positive work attitudes and engage in positive work behaviors (Edwards and Shipp, 2007; Kim et al., 2017). In the organization, the leader’s role is generally viewed as setting goals, solving problems, and providing help, and leaders thus are expected to play a critical role in fulfilling employees’ needs during work (Zaccaro, 2014).

Combining needs-supplies fit theory and the LMX research, this study includes employees’ expectation of feedback quality in perceiving leader feedback giving and posits that congruence between expected feedback quality and delivered feedback quality results in high-quality LMX. On the one hand, LMX evolves in response to the exchanges between a leader and an employee where each party provides the other with what the other party attaches importance to (Park et al., 2015). Previous research suggests that leader behaviors are important drivers of LMX (Gottfredson et al., 2020), and one of leader behaviors that received much attention is feedback giving. Employees expect leaders to provide them with effective feedback concerning their jobs. When employees’ expectations are met by leaders, they are likely to sustain a favorable exchange relationship with their leaders and thus perceive a high-quality LMX. Furthermore, LMX is characterized by mutual understanding, trust, and respect (Liden and Maslyn, 1998). Congruence between expected feedback quality and delivered feedback quality will cause employees to feel valued, and induce more positive feelings toward the leader, leading to higher quality LMX and increasing the likelihood of employee reward, such as improving employee performance (Marstand et al., 2017).

As for incongruences, there are two situations. Based on the literature of needs-supplies fit theory (Edwards, 1996), we propose that both incongruences getting lower-quality feedback than one expects (undersupply) and getting higher-quality feedback than one expects (oversupply) are problematic. First, undersupply (i.e., expected feedback quality > delivered feedback quality) represents that employee expectations are not being fulfilled, which means that leaders are not giving them what they want to form a virtuous exchange relationship, and thus LMX will be violated. Second, oversupply (i.e., delivered feedback quality > expected feedback quality) intuitively seems to be a benefit to the employee. However, studies have revealed that oversupply can develop a stressful situation for employees. For example, Rupprecht et al. (2016) found when employees received sensitivity from leaders above what individuals expect, employees’ other work-related needs may be hindered. Harris and Kacmar (2006) also noticed that when employees receive an excessive amount of trust and emotional support from the leader could also cause job tension. For this study, feedback is a job component that needs to be digested and absorbed by employees to improve their job. Thus, oversupply in the case that delivered feedback quality by leaders excess expected feedback quality by employees might cause a stressor for employees. It can create a feeling that employees need to accomplish more tasks by themselves to form a balanced exchange with their leaders. Thus:

*Hypothesis 1*: Compared to inconsistency between expected feedback quality and delivered feedback quality, the more consistent expected feedback quality and delivered feedback quality are, the higher LMX is. That is, the degree of incongruence between expected feedback quality and delivered feedback quality is negatively related to LMX, such as when expected (delivered) feedback quality exceeds delivered (expected) feedback quality, employees will experience a decrease in LMX.

Furthermore, related to this hypothesis is that when expected feedback quality and delivered feedback quality are in congruence, employees will perceive higher levels of LMX. Namely, greater congruence between expected feedback quality and delivered feedback quality brings higher LMX while greater incongruence is related to lower LMX. However, as proposed by Edwards (2002), when exploring the implications of fitting variables, it is necessary to consider their fitness level. Thus, this study posits that congruence between expected feedback quality and delivered feedback quality at a higher level may be more meaningful than at a lower level. On the one hand, high expectations are accompanied by a sense of eagerness and excitement. After the expectation is satisfied, those eagerness and excitement can be released smoothly, and individuals can obtain greater satisfaction. Besides, research has found that employees tend to expect high feedback quality from leaders and benefit more from receiving such feedback (Whitaker and Levy, 2012; Wang et al., 2015). Steelman et al. (2004) also believed that employees could expect that high feedback quality can create higher positive performance. Thus, we expect that meeting employees’ high feedback quality expectations may lead to higher positive outcomes because higher feedback quality allows employees to perform better in the future. Also, employees who receive high levels of feedback quality fulfillment are more likely to view this fulfillment as a kind of incentive than receiving low levels of fulfillment, because the leader is meeting their more needs and thus likely developing more positive behaviors. Thus, we expect that those who expect and get a higher level of feedback quality from leaders would experience a higher level of LMX. As such, we hypothesized:

*Hypothesis 2*: Congruence at higher levels of feedback quality (i.e., when expected feedback quality and delivered feedback quality are both high) is related to higher levels of LMX as compared with congruence at lower levels of feedback quality.

### The mediating role of LMX

Research on LMX suggests that high-quality LMX increasingly engenders feelings of mutual obligation and reciprocity, which triggers employees’ positive work behavior (Dulebohn et al., 2012). Previous research has consistently shown that LMX is positively related to task performance and OCB (Breevaart et al., 2015; Anand et al., 2018; Che et al., 2021; Park et al., 2022). Task performance refers to “the basic required duties of a particular job.” (Ng and Feldman, 2010, p. 1223). Employees in a high-quality LMX should exhibit better task performance because they receive more valuable resources such as support, and direction from leaders, which are functional to achieve work goals (Loi et al., 2011). OCB refers to the behavior that “is either not required by law or is essentially unenforceable by the usual incentives or sanctions” (Smith et al., 1983, p. 654). Since leaders are generally considered as the representative of their organizations, employees perceiving a high-quality LMX may be motivated to devote extra efforts to the organization (Xu et al., 2012).

In the context of needs-supplies fit, we argue that LMX is particularly sound for the indirect relationship between congruence between expected feedback quality and delivered feedback quality and task performance and OCB. The central rationale underlying this relationship is that fulfillment of work expectations evokes a better LMX relationship, which sparks behavioral reciprocity from employees through making more efforts in in-role performance or extra-role performance. Specifically, LMX has been proposed as an important mechanism between person-supervisor fit on employee work attitudinal or behavioral outcomes (e.g., Van Vianen et al., 2011; Marstand et al., 2017; Khorakian and Sharifirad, 2019). For example, Marstand et al. (2017) found that LMX mediates the relationship between the fulfillment of different work values and employee work outcomes. Parent-Rocheleau et al. (2020) found that LMX plays a mediating role in the relationship between leader-follower (dis)similarity in psychological capital and employee outcomes. Combined with LMX literature, when leaders’ delivered feedback quality meets employees’ expectations, employees could get valuable information for attitudes or behaviors desired by the organization, the comparative level at which he or she is meeting job standards, and job advancement in this organization (Ashford et al., 2016), leading to a high-quality LMX perception. Then employees have sufficient resources to perform their jobs well or reciprocate leaders by improving task performance or exhibiting more OCB.

However, when it comes to the indirect effects of incongruences on employee performance, we need to distinguish these two cases (i.e., task performance or OCB). In terms of the indirect effect of incongruences on employee task performance *via* LMX, we argue that LMX may not significantly mediate the relationship between incongruences and employee task performance because of the inherent value of feedback in improving task performance. Specifically, when expected feedback quality exceeds delivered feedback quality, employees experience a sense of disappointment resulting in the low perception of LMX. But, due to employees did not receive effective feedback to improve their task performance; their task performance may not change. Also, when delivered feedback quality exceeds the expected feedback quality, there is a perception of job pressure, symbolized by additional work tasks (Sonnentag and Fritz, 2015), which may also result in a negative evaluation of the relationship with the leader (i.e., low LMX). Although employees have the resource (i.e., high-quality feedback) to improve their work at this time, they may not be motivated enough to do so because of a negative perception of LMX. Therefore, their job performance at this time will not improve either. However, we believe that LMX can effectively mediate the relationship between incongruences and employee OCB. OCB serves as an extra-role behavior, which is not part of the job description (Chen et al., 2014), thus a decrease in LMX due to incongruences may further cause a decrease in OCB. Accordingly, we propose the following hypothesis:

*Hypothesis 3*: LMX mediates the relationship between (in)congruence between expected feedback quality and delivered feedback quality and task performance (H3a) and OCB(H3b). Specifically, greater congruence between expected feedback quality and delivered feedback quality will be positively related to employee task performance and OCB via LMX, while increased incongruence (in either direction) between expected feedback quality and delivered feedback quality will negatively be related to employee OCB via LMX.

### The moderating role of learning goal orientation

As suggested by the needs-supplies fit theory, the impact of the degree of fit on individual outcomes would be dependent on how much employees valued the supplies (Edwards and Shipp, 2007). Here we focus on learning goal orientation, which is defined as “in which individuals seek to increase their competence, to understand or master something new” (Dweck, 1986, p. 1040). Since individuals with learning goal orientation attach more importance to leaders’ feedback (VandeWalle, 2003; Vandewalle et al., 2019), we expect that learning goal orientation will amplify the positive effects of congruence between expected feedback quality and delivered feedback quality.

Specifically, individuals with higher learning goal orientation focus on their ability development (VandeWalle et al., 2019). Given that feedback is beneficial for promoting the development of ability (VandeWalle, 2003); employees would pay more attention to whether feedback from leaders meets their requirements. Compared with high learning goal-oriented individuals who are concerned about the value of receiving feedback, low learning goal-oriented individuals care more about the cost of receiving feedback such as receiving unfavorable appraisals (VandeWalle, 2003; Park et al., 2007). Thus, for employees with high learning goal orientation, once delivered feedback from the leader meets their expectations, they will perceive more support and relational reciprocity from the leader. In contrast, low learning goal-orientated employees may shrink the strength of the relationship between congruence between expected feedback quality and delivered feedback quality and LMX.

Moreover, the relationship between incongruences and LMX may also be influenced by learning goal orientation. When expected feedback quality exceeds delivered feedback quality, individuals with a high learning goal orientation will experience greater dissatisfaction compared with low learning goal orientation. The negative effects of incongruence on LMX will be exacerbated. However, when delivered feedback quality exceeds expected feedback quality, individuals with a high learning goal orientation rather than a low one may be pleasantly surprised because of receiving more resources than expected that can further improve their work. The negative effect of incongruence (i.e., delivered feedback quality exceeds expected feedback quality) on LMX will be reduced. We, therefore, hypothesize that:

*Hypothesis 4*: Learning goal orientation moderates the effect of (in)congruence between expected feedback quality and delivered feedback quality on LMX. Specifically, the positive effect of congruence between expected feedback quality and delivered feedback quality on LMX is stronger, while the negative effect of incongruence (delivered feedback quality < expected feedback quality) on LMX is stronger among employees with higher (vs. lower) learning goal orientation.

Combining H3 and H4, we further propose the moderated mediation models. Specifically, employees with a high learning goal orientation rather than a low learning goal orientation value more leaders’ feedback, and thus congruence between expected feedback quality and delivered feedback quality will result in a higher quality of LMX. Such LMX will be reciprocated by employees putting more effort into task performance and OCB. But, for high learning goal-oriented individuals rather than low, when delivered feedback quality exceeds expected feedback quality, it is more possible for them to regard oversupply as kind attention from the leader instead of a burden. Thus, high learning goal-oriented individuals will put high-quality feedback for work improvement. Moreover, high learning goal-oriented individuals rather than low are devoted to improving their work. Thus, we propose undersupplying feedback quality (delivered feedback quality < expected feedback quality) may not demotivate them to do it. On the other hand, employees with a high learning goal orientation rather than a low learning goal orientation are more unacceptable that delivered feedback quality is less than expected feedback quality, so the effect of incongruence (delivered feedback quality < expected feedback quality) on LMX can be more negative, and employees thus show less reciprocal behavior (i.e., OCB). However, oversupply (delivered feedback quality > expected feedback quality) also provides additional workload to employees. Individuals with a high learning goal orientation have less energy to devote to extra-role behavior (i.e., OCB). Thus, the negative effect of incongruence (delivered feedback quality > expected feedback quality) on OCB *via* LMX maybe strengthen by high learning goal orientation rather than low. We propose the following hypothesis:

*Hypothesis 5*: Learning goal orientation moderates the indirect effect of (in)congruence between expected feedback quality and delivered feedback quality on task performance (H5a), and OCB (H5b) via LMX. Specifically, the positive indirect effects of congruence between expected feedback quality and delivered feedback quality on employee task performance and OCB are stronger, while the negative indirect effects of incongruence (delivered feedback quality < expected feedback quality) on OCB are stronger among employees with higher (vs. lower) learning goal orientation.

## Method

### Sample and procedure

The samples of this study are recruited from four organizations located in Northern China, which are embedded in the industry of manufacturing. Questionnaires were collected from 386 employees. We first connected managers of their human resource management departments and explained our research aims and procedures. After getting their permission, we distributed our electronic questionnaire through WeChat, a widely used social application in China, with the help of the human resource management department. We promised that the participation is voluntary and confidential, and 5 RMB would be paid after each time they complete the survey.

To reduce the potential effects of common method bias, data were collected at two-time points. In the first wave, participants were asked to report their expected feedback quality, learning goal orientation, and demographic information. A total of 267 employees completed the first survey with a 69.17% response rate. At the second wave, 1 month later, we sent another survey to those employees and asked them to provide assessments about the received feedback quality, LMX, task performance, and OCB. To match participants’ responses, a subject ID number was assigned, and we asked participants to fill in their numbers in the first and second surveys.

After eliminating mismatches and incomplete questionnaires, 226 matched data were included in the final sample, with a total 58.55% response rate. The average age for participants was 30.77 years (*SD* = 8.15). 60.09% of the respondents are male. Education levels for participants of master or above, bachelor, junior college, and junior college below were 44.64, 43.78, 4.29, and 7.30%, respectively. The average organizational tenure was 46.02 months (*SD* = 57.28). Participants’ average dyadic tenure with leaders was 35 months (*SD* = 46.35). They held a wide range of positions in the organization: rank-and-file employee (52.79%), general manager (18.02%), middle manager (15.02%), and senior manager (14.16%), and are from various organizational departments: accounting/ legal and audit (18.45%), general management (19.74%), marketing (15.02%), operation information (36.48%), quality control (0.73%), and R&D (0.34%).

### Measures

Following the translation-back-translation procedure (Brislin, 1980), the authors who are all proficient in both Chinese and English translate the established English measures into Chinese. Except for control variables, all scales were measured on a five-point Likert-type scale ranging from 1 (strongly disagree) to 5 (strongly agree).

#### Congruency between expected feedback quality and delivered feedback quality

Five-item feedback quality scale of Steelman et al. (2004) was used to assess expected feedback quality and delivered feedback quality. A sample item of expected feedback quality is “The job feedback I expect from my direct leader will be helpful.” A sample item of experienced feedback quality is “The job feedback I obtain from my direct leader is helpful.” Consistent with previous studies (Parent-Rocheleau et al., 2021), congruence was explained by polynomial regression and response surface analysis techniques (α = 0.87, 0.91, respectively).

#### Leader-member exchange

Leader-member exchange was measured with the seven-item scale developed by Graen and Uhl-Bien (1995). One sample item is “My manager understands my problems and needs.” (α = 0.93).

#### Learning goal orientation

Learning goal orientation was measured by VandeWalle (1997) with a five-item scale. An example item is “I often look for opportunities to develop new skills and knowledge.” (α = 0.90).

#### Task performance

Employees rated their task performance *via* a three-item scale adapted from Farh et al. (1991). An example item is “What do you think of your quality of work? In other words, are your work outcomes perfect, free of error, and high accuracy?” (α = 0.86).

#### Organizational citizenship behavior

We used a 16-item survey developed by Smith et al. (1983). An example item is “I help others who have heavy workloads.” (α = 0.93).

#### Control variables

Age, gender, education, and organizational tenure were included as control variables because they have been shown to affect task performance and OCB (Gerstner and Day, 1997; Ng and Feldman, 2010). Moreover, to explain the potential familiarity effect, dyadic tenure between the leader and the employee was also included in the analyses (Van Vianen et al., 2011). As recommended by Becker et al. (2016), hypotheses were tested with and without these control variables. As the results show the same patterns, to report briefly, the findings shown in the following section were without control variables.

### Analytic strategy

In line with previous studies (e.g., Guan and Frenkel, 2021; Parent-Rocheleau et al., 2021; Zhang et al., 2022), we utilize polynomial regression techniques and response surface analysis to test hypotheses with the help of software SPSS 22.0 and Microsoft Excel. To effectively reduce multicollinearity and facilitate the interpretation of the results, this study centralizes the expected feedback quality and the delivered feedback quality before calculating the polynomial regression (Aiken et al., 1991). Then we calculate the square term and their interaction. The basic polynomial regression equation is Y = a_0_ + a_1_EFQ + a_2_DFQ + a_3_EFQ^2^ + a_4_EFQ^*^DFQ + a_5_DFQ^2^ + e, where a represents the regression coefficient for each variable. EFQ represents expected feedback quality, while DFQ represents delivered feedback quality. EFQ^2^ and DFQ^2^ represent the squared terms of expected feedback quality and delivered feedback quality, respectively. EFQ^*^DFQ is an interaction between expected feedback quality and delivered feedback quality.

## Results

### Confirmatory factor analyses

The MPLUS 7.0 was used to conduct confirmatory factor analyses. Following the recommendation from Little et al. (2002), we conducted item parcels for all variables, and 18 parcels were generated for six variables. The results of the data analysis show that the six-factor model including expected feedback quality, delivered feedback quality, LMX, learning goal orientation, task performance, and OCB (χ^2^ = 140.53, df = 120, CFI = 0.99, TLI = 0.99, RMSEA = 0.03, SRMR = 0.03) fits the data better than all alternative models, which means that the measures used in this study captured distinct construct (Brown and Moore, 2012; Lin et al., 2021). The Durbin-Watson statistic values of all regression models are provided in Table 1, ranging from 1.84 to 2.12. Thus, multicollinearity is not an issue in our study (Parent-Rocheleau et al., 2020).

**Table 1:** Descriptive statistics and variable correlations.

	Mean	SD	1	2	3	4	5	6	7	8	9	10
1. Expected feedback quality	4.38	0.59	(0.87)									
2. Delivered feedback quality	3.94	0.79	0.41^**^	(0.91)								
3. LMX	3.87	0.82	0.32^**^	0.47^**^	(0.93)							
4. Learning goal orientation	3.94	1.03	0.12	0.25^**^	0.26^**^	(0.90)						
5. Task performance	4.10	0.64	0.27^**^	0.38^**^	0.41^**^	0.32^**^	(0.86)					
6. OCB	4.05	0.62	0.25^**^	0.44^**^	0.51^**^	0.36^**^	0.61^**^	(0.93)				
7. Gender	1.40	0.49	0.05	−0.02	0.07	0.02	−0.00	0.05				
8. Age	30.77	8.15	0.04	−0.02	0.09	−0.12	0.04	−0.00	0.05			
9. Education	3.30	0.80	−0.08	0.08	0.01	0.05	0.02	0.09	−0.11	−0.17^*^		
10. Tenure	46.02	57.28	−0.03	0.08	0.07	0.16^*^	0.06	0.12	0.10	−0.19^**^	0.57^**^	
11. Dyadic tenure	35.00	46.35	−0.05	0.09	0.03	0.15^*^	0.05	0.12	0.18^**^	−0.10	0.33^**^	0.67^**^

*N* = 226; ^*^p < 0.05; ^**^p < 0.01; Gender was coded as “1” for male and “2” for female; Education was coded as “1” for junior college below, “2” for junior college, “3” for bachelor, and “4” for master or above.

### Descriptive statistics

The means, standard deviations, correlations, and Cronbach’s alphas for all the variables are reported in Table 2. All variables have acceptable internal consistency alphas of above 0.70. Expected feedback quality was significantly related to LMX (*r* = 0.32, *p* < 0.01), employee task performance (*r* = 0.27, *p* < 0.01), and OCB (*r* = 0.25, *p* < 0.01). Delivered feedback quality was significantly related to LMX (*r* = 0.47, *p* < 0.01), employee task performance (*r* = 0.38, *p* < 0.01), and OCB (*r* = 0.44, *p* < 0.01). LMX was significantly associated with employee task performance (*r* = 0.41, *p* < 0.01), and OCB (*r* = 0.51, *p* < 0.01). However, the control variables were not significantly related to LMX and employee performance. Thus, following recommendation of Becker et al. (2016), control variables are not included in hypothesis testing.

**Table 2:** Confirmatory factor analyses results.

Model	χ^2^	df	Δχ^2^	Δdf	CFI	TLI	RMSEA	SRMR
1. EFQ, DFQ, LMX, LGO, TP, OCB	140.53	120	-	-	0.99	0.99	0.03	0.03
2. EFQ + DFQ, LGO, LMX, TP, OCB	420.74	125	280.21	5	0.90	0.88	0.10	0.08
3. EFQ + DFQ + LGO, LMX, TP, OCB	876.44	129	735.91	9	0.75	0.71	0.16	0.13
4. EFQ + DFQ + LGO, LMX, TP + OCB	1038.49	132	897.96	12	0.70	0.65	0.17	0.14
5. EFQ + DFQ + LGO, LMX + TP + OCB	1447.61	134	1307.08	14	0.56	0.50	0.21	0.15
6. EFQ + DFQ + LMX + LGO + TP + OCB	1772.79	135	1632.26	15	0.46	0.38	0.23	0.14

EFQ, expected feedback quality; DFQ, delivered feedback quality; LMX, leader-member exchange; LGO, learning goal orientation; TP, task performance; OCB, and organizational citizenship behavior.

### Hypotheses testing

**Figure 2:**
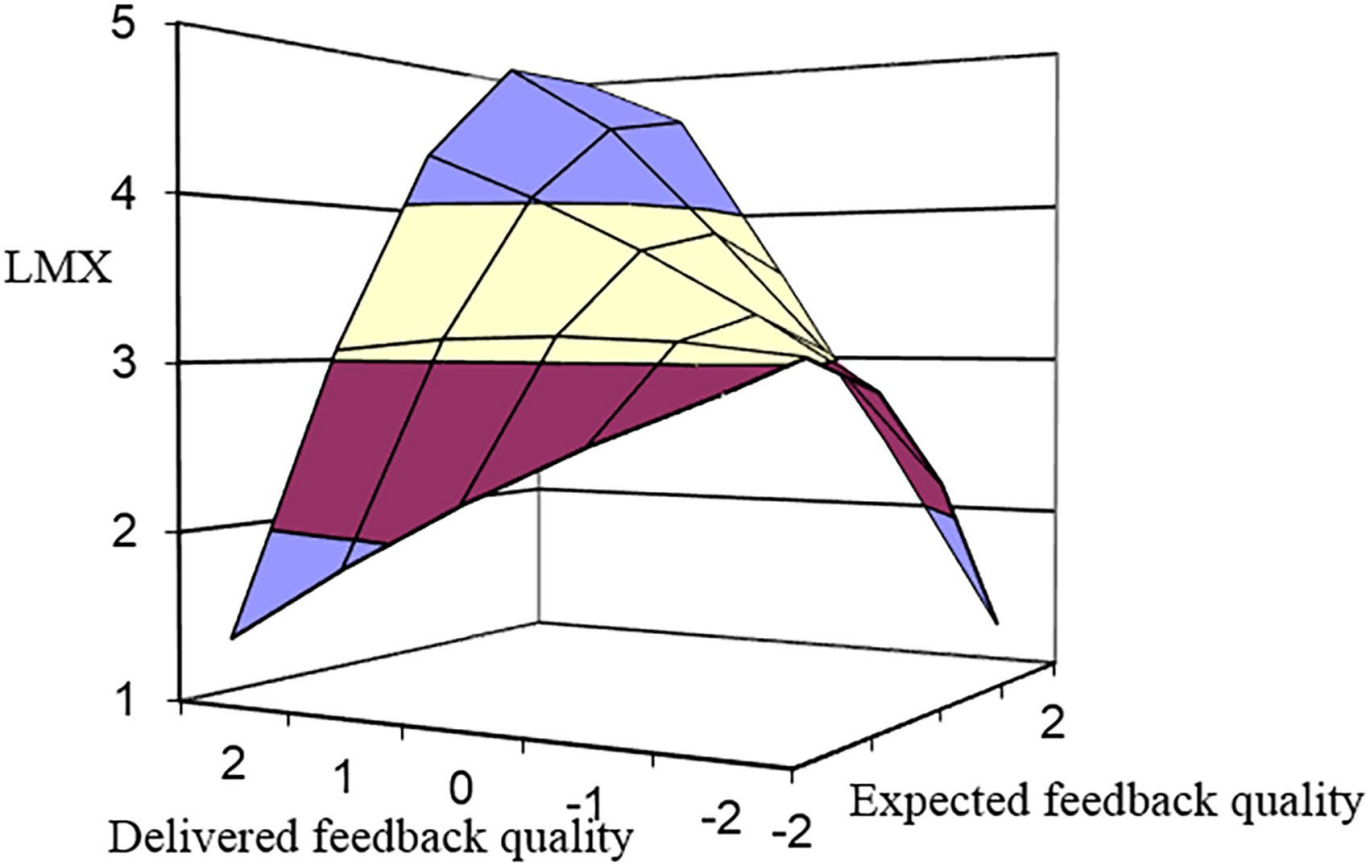
Direct effect of expected-delivered feedback quality congruence on leader-member exchange (LMX).

To test Hypothesis 1, we first entered expected feedback quality (EFQ) and delivered feedback quality (DFQ) into the regression equation to test how they have a linear impact on the LMX (Model 1). Then, we entered EFQ^2^, the interaction term EFQ × DFQ, and DFQ^2^ into the equation to test the interaction effect (Model 2). As shown in Table 1, the Δ*R*^2^ of Model 2 after putting the quadratic and interaction terms has a significant incremental significance compared with Model 1 (M2: *ΔR^2^* = 0.03, *p* < 0.05). It indicates that the polynomial effects (i.e., the effect of quadratic and interaction terms between expected feedback quality and delivered feedback quality) predicts LMX beyond the respective baseline effects of expected feedback quality and delivered feedback quality (Parent-Rocheleau et al., 2020). The results in Table 3 show that coefficient b_1_ is significant and negative (*b_4_* = −0.59, *p* < 0.01), showing that moving along the incongruence line from the left to right to the congruence line, LMX increases as delivered feedback quality increase toward the expected feedback quality, after which LMX decreases. Hypothesis 1, which predicted that congruence between expected feedback quality and delivered feedback quality would positively influence employees’ perception of LMX, is thus supported. Moreover, the significance of the slope of congruence (*b_1_* = 0.72, *p* < 0.01) indicates that there is a significant difference in LMX between a situation of congruence between expected feedback quality and delivered feedback quality of high levels and a situation of congruence between expected feedback quality and delivered feedback quality of low levels, and LMX is higher when congruence between expected feedback quality and delivered feedback quality is at high levels. This is consistent with our expectations. Thus, Hypothesis 2 is supported. Figure 2 also shows that LMX increases following a linear way along the diagonal line of congruence and is higher in the high-high congruence situation compared with the low-low congruence situation.

**Table 3:** The polynomial regression analyses results.

	LMX				Task performance				OCB			
	M1	M2	M3	M4	M5	M6	M7	M8	M10	M11	M12	M13
Constant	3.67^**^	3.68^**^	3.69^**^	3.74^**^	3.91^**^	3.08^**^	3.88^**^	3.38^**^	3.96^**^	3.03^**^	3.96^**^	3.43^**^
EFQ	0.23^*^	0.34^**^	0.35^**^	0.36^**^	0.11	0.03	0.13	0.08	0.12	0.03	0.13	0.08
DFQ	0.45^**^	0.38^**^	0.33^**^	0.25^**^	0.34^**^	0.25^**^	0.20^**^	0.17^*^	0.31^**^	0.21^**^	0.17^**^	0.14^*^
EFQ^2^		−0.19	−0.17	−0.50^**^	−0.09	−0.05	−0.06	0.00	−0.27^**^	−0.22^*^	−0.32^*^	−0.24
EFQ × DFQ		0.37^**^	0.37^**^	0.44^**^	0.04	−0.05	0.04	−0.02	0.32^**^	0.22^*^	0.36^**^	0.30^**^
DFQ^2^		−0.03	−0.04	−0.10	0.08	0.08	0.02	0.03	0.04	0.04	−0.02	−0.01
LGO			0.12^*^	0.28^**^			0.11	0.07			0.18^**^	0.14^**^
EFQ × LGO				0.20			0.12	0.09			0.08	0.05
DFQ × LGO				0.19^*^			0.25^**^	0.22^**^			0.16^**^	0.14
EFQ^2^ × LGO				−0.45^*^			−0.02	0.04			−0.26	−0.20
EFQ × DFQ × LGO				0.34^**^			0.07	0.03			0.27^**^	0.22^**^
DFQ^2^ × LGO				−0.00			0.12^*^	0.12^*^			0.10^*^	0.10^*^
LMX						0.22^**^		0.13^*^		0.25^**^		0.14^**^
*R^2^*	0.25	0.28	0.30	0.46	0.17	0.23	0.31	0.32	0.25	0.34	0.41	0.43
Δ*R^2^*		0.03^*^	0.02^**^	0.16^**^		0.06^**^	0.14^**^	0.01^*^		0.09^**^	0.16^**^	0.02^**^
*F-statistic*	36.37^**^	17.27^**^	15.80^**^	16.70^**^	9.13	10.83	8.58^**^	8.42^**^	14.73^**^	18.46^**^	13.32^**^	13.19^**^
*D-W*	1.86	1.91	1.94	2.12	1.84	1.94	1.87	1.93	1.85	1.88	1.85	1.84

*N* = 226; ^*^p < 0.05. ^**^p < 0.01; EFQ, expected feedback quality; DFQ, delivered feedback quality; and LGO, learning goal orientation.

#### Mediation effects test

**Figure 3:**
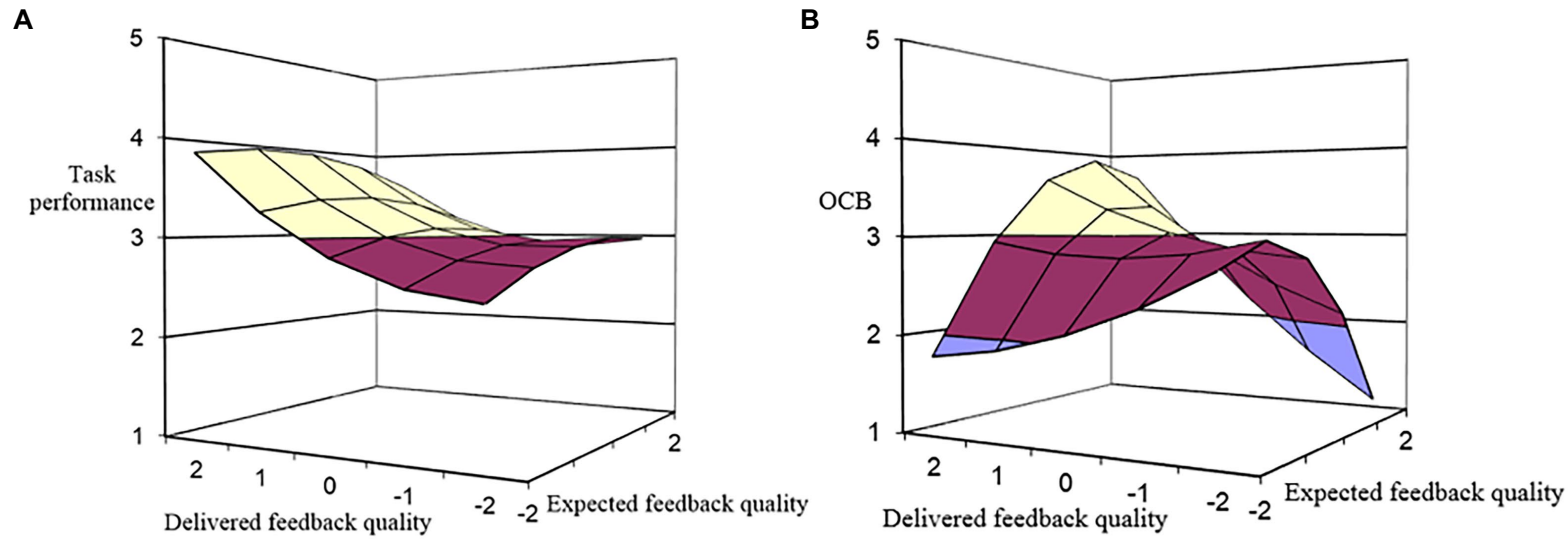
Indirect effects of expected-delivered feedback quality congruence on **(A)** task performance and **(B)** OCB (*via* LMX).

To test the indirect effect of (in)congruence between expected feedback quality and delivered feedback quality on task performance through LMX, we added LMX to the model regressing task performance on the five polynomial regression terms. As shown in Table 1, the influence of LMX on task performance was found to be significant beyond the effect of polynomial terms (M6: *β* = 0.22, *p* < 0.01), and its addition increases the explained variance of task performance (*ΔR^2^* = 0.06, *p* < 0.01). As shown in Table 3, the b_1_ coefficient indicates a significant effect of congruence between expected feedback quality and delivered feedback quality on task performance (*b_1_* = 0.29, *p* < 0.01). Figure 3A describes this effect and shows that the indirect effect of congruence between expected feedback quality and delivered feedback quality on task performance *via* LMX is linear, such that when the congruence is at higher levels, employees exhibit higher task performance. However, as shown in Table 3, the indirect effect of incongruence between expected feedback quality and delivered feedback quality on task performance *via* LMX is not significant (*b_2_* = −0.01, *p* > 0.05; *b_3_* = −0.22, *p* > 0.05; *b_4_* = 0.08, *p* > 0.05). Thus, Hypothesis 3a is supported.

Similarly, to test the indirect effect of (in)congruence between expected feedback quality and delivered feedback quality on OCB through LMX, we added LMX to the model regressing OCB on the five polynomial regressions terms. As shown in Table 1, the effect of LMX on OCB is significant beyond the effect of polynomial terms (M10: *β* = 0.25, *p* < 0.01), and its addition increases the explained variance of OCB (*ΔR^2^* = 0.09, *p* < 0.01). As shown in Table 3, The b_1_ and b_4_ coefficients indicate a significant effect of congruence between expected feedback quality and delivered feedback quality on OCB (*b_1_* = 0.24, *p* < 0.01; *b_4_* = −0.40, *p* < 0.05). Figure 3B describes this effect and shows that the indirect effect of congruence between expected feedback quality and delivered feedback quality on OCB is significantly positive, such that moving along the incongruence line from the left to right to the congruence line, OCB increases as delivered feedback quality increase toward expected feedback quality, after which OCB decreases. And, when the congruence is at higher levels, employees engage in more OCB. Moreover, the significant b_4_ coefficient and Figure 3B also show that regardless of whether expected feedback quality exceeds delivered feedback quality or vice versa, OCB always decreases as the difference between the two becomes greater. Thus, Hypothesis 3b is supported.

#### Moderation effects test

**Figure 4:**
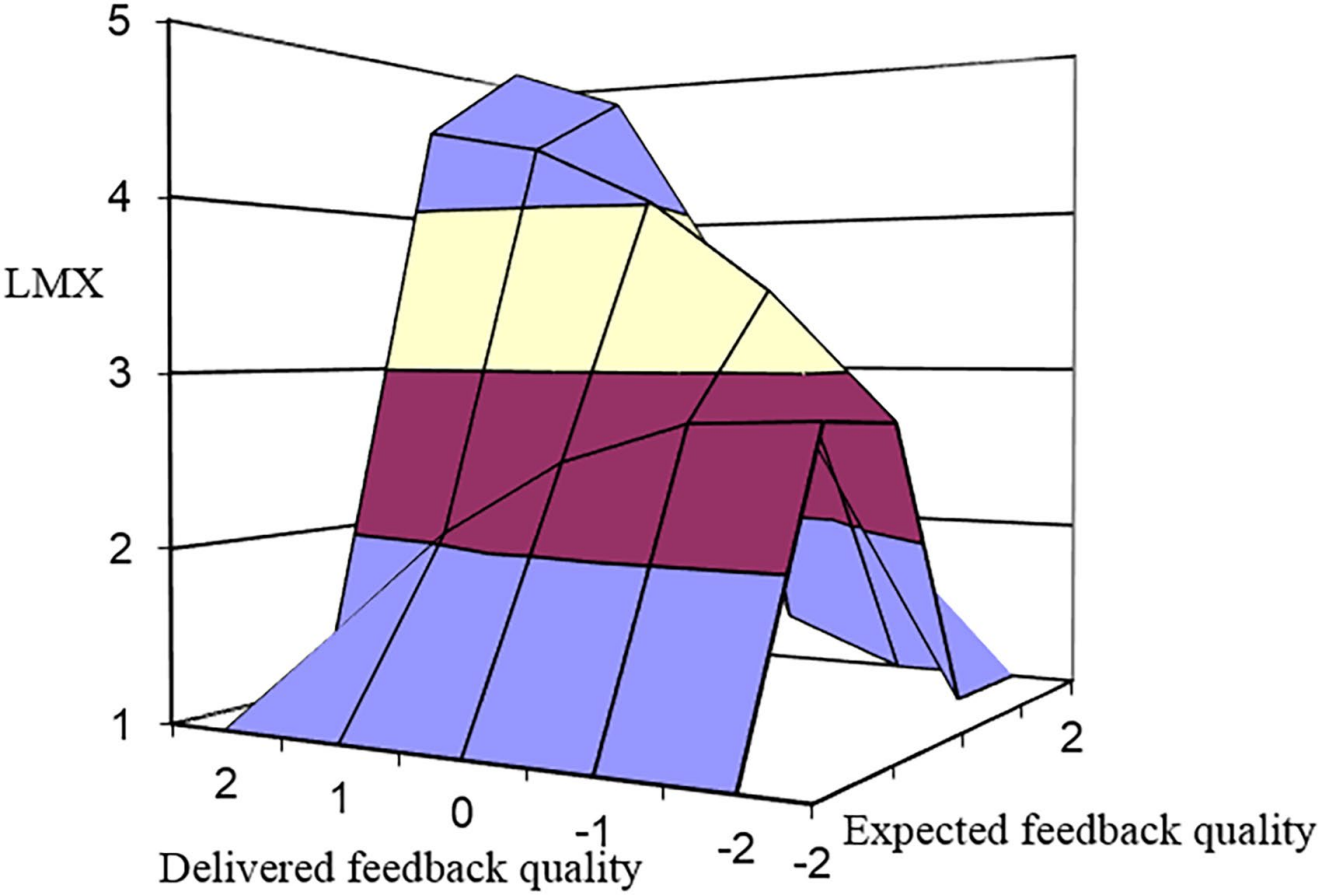
Direct effect of expected-delivered feedback quality congruence on LMX at a high level of learning goal orientation.

Hypothesis 3 proposes that the effect of congruence between expected feedback quality and delivered feedback quality on LMX is stronger for employees with high learning goal orientation. The results presented in Table 3 show that the interaction of the polynomial terms with the learning goal orientation significantly explains the variance of LMX, which exceeds the main influence of the learning goal orientation itself (M4: *ΔR^2^* = 0.16, *p* < 0.01). To clearly show the moderating effect of learning goal orientation, one standard deviation above and below the mean of learning goal orientation was taken into moderation analyses. As shown in Table 3 and Figure 4, the positive effect of congruence between expected feedback quality and delivered feedback quality on LMX is significantly stronger when learning goal orientation is higher (*b_1_* = 1.01, *p* < 0.01) than lower (*b_1_* = 0.21, *p* < 0.05). However, when learning goal orientation is high rather than low, the b_4_ coefficient is significant (*b_1_* = −1.85, *p* < 0.01) and indicates that moving along the incongruence line from the left to right to the congruence line, LMX increases as delivered feedback quality increase toward expected feedback quality, after which LMX decreases. Namely, whether the delivered feedback quality is greater than expected feedback quality or less than expected feedback quality, LMX keeps decreasing as the difference between the two becomes larger. Thus, hypothesis 4 receives partial support.

#### Moderated mediation effects test

**Figure 5:**
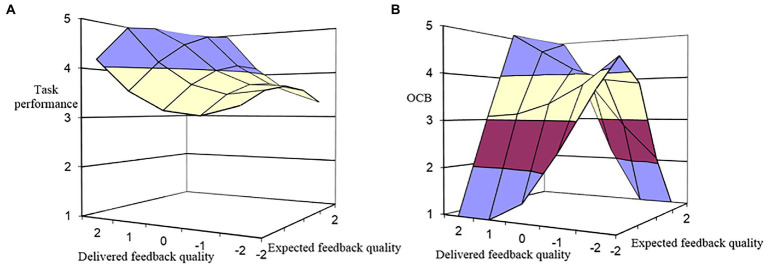
Indirect effects of expected-delivered feedback quality congruence on **(A)** task performance and **(B)** OCB (*via* LMX) at a high level of learning goal orientation.

Hypotheses 5a,b propose that the positive indirect effects of congruence between expected feedback quality and delivered feedback quality on employee task performance and OCB are stronger, while the negative indirect effects of incongruence (delivered feedback quality < expected feedback quality) on employee task performance and OCB are stronger among employees with higher (vs. lower) learning goal orientation. As shown in Table 1, the indirect impact of (in)congruence between expected feedback quality and delivered feedback quality on task performance is moderated by learning goal orientation, because the addition of the mediator (LMX) further increases the explained variance of task performance, which exceeds the impact of the interaction terms (M8: *ΔR^2^* = 0.13, *p* < 0.05). The response surface coefficients in Table 3 show that when employees are in high learning goal orientation, the indirect effect of congruence between expected feedback quality and delivered feedback quality on task performance is significant and stronger (*b_1_* = 0.71, *p* < 0.01). These findings are shown in Figure 5A, depicting that in the case of high learning goal orientation task performance increases positively, and task performance is higher when expected feedback quality is consistent with delivered feedback quality at high levels rather than at low levels. However, the indirect effect incongruence between expected feedback quality and delivered feedback quality on task performance is not significant no matter whether learning goal orientation is high (*b_2_* = 0.16, *p* > 0.05; *b_3_* = −0.21, *p* > 0.05; *b_4_* = −0.11, *p* > 0.05) or low (*b_2_* = −0.18, *p* > 0.05; *b_3_* = 0.06, *p* > 0.05; *b_4_* = −0.11, *p* > 0.05). H5a is thus partially supported.

Similarly, the result of the moderating role of learning goal orientation in the indirect impact of (in)congruence between expected feedback quality and delivered feedback quality on OCB (H5b) is also shown in Table 1. The addition of the mediator (LMX) also further increases the explained variance of OCB beyond the influence of the interaction terms (M12: *ΔR^2^* = 0.14, *p* < 0.01). The response surface coefficients in Table 3 show that when employees report high learning goal orientation (*b_1_* = 0.55, *p* < 0.01), the indirect effect of congruence is significant and stronger. Figure 5B depicts the indirect effect of (in)congruence between expected feedback quality and delivered feedback quality on OCB *via* LMX, describing that when expected feedback quality is consistent with delivered feedback quality at a high level than at a low level, OCB increases positively, and OCB is higher. Moreover, along the incongruence line from left to right to the congruence line, OCB increases as delivered feedback quality increase toward expected feedback quality, after which OCB decreases (*b_4_* = −1.14, *p* < 0.01). It indicates that whether expected feedback quality exceeds delivered feedback quality or *vice-versa*, OCB becomes lower as the difference between the two becomes greater. These results provide support for H5b (Table 4).

**Table 4:** Response surface coefficients.

	LMX			Task performance			OCB		
	Overall	Low LGO	High LGO	Overall	Low LGO	High LGO	Overall	Low LGO	High LGO
*b*_1_	0.72^**^	0.21^*^	1.01^**^	0.29^**^	−0.04	0.71^**^	0.24^**^	0.06	0.55^**^
*b*_2_	0.15	−0.04	−0.28	−0.01	−0.18	0.16	0.05	−0.09	0.14
*b*_3_	−0.04	0.10	0.11	−0.22	0.06	−0.21	−0.18	0.04	−0.13
*b*_4_	−0.59^**^	−0.22	−1.85^**^	0.08	−0.11	−0.06	−0.40^*^	−0.26	−1.14^**^

*N* = 226; ^*^p < 0.05. ^**^p < 0.01; LGO, learning goal orientation; *b*_1_ = (*a*_1_ + *a*_2_), *b*_2_ = (*a*_3_ + *a*_4_ + *a*_5_), *b*_3_ = (*a*_1_ − *a*_2_), *b*_4_ = (*a*_3_ − *a*_4_ + *a*_5_); The *b*_1_ and *b*_2_ represent separately the slope and curvature of congruence, the *b*_3_ and *b*_4_ represent separately the slope and curvature of incongruence.

## Discussion

As previous research found mixed relationships between leader feedback quality and job performance, this study applied polynomial regression to give a more nuanced exploration of how (in)congruence between expected feedback quality and delivered feedback quality affects job performance. According to needs-supplies fit theory and LMX literature, this study adopted data collected at two points to examine whether (in)congruence between expected feedback quality and delivered feedback quality influences job performance through LMX. Results show that congruence between expected feedback quality and delivered feedback quality is positively related to LMX. LMX mediates the relationship between congruence between expected feedback quality and delivered feedback quality and task performance and OCB.

Furthermore, we find that incongruence between expected feedback quality and delivered feedback quality harms LMX (*b_4_* = −0.59, *p* < 0.01 in Table 3) and OCB (*b_4_* = −0.40, *p* < 0.05 in Table 3). The results indicate that regardless of whether leaders’ delivered feedback quality is more or less than employees’ expected feedback quality, LMX and OCB would be decreased. Notably, the indirect effect of incongruence between expected feedback quality and delivered feedback quality on task performance is not significant (*b_4_* = 0.08, *p* > 0.01 in Table 3). The underlying reason may be that when expected feedback quality exceeds delivered feedback quality, leaders could not provide necessary job-related resources to improve task performance; when leaders deliver higher feedback quality than expected, employees may feel disturbed and overloaded (Lambert et al., 2003; Wong and Giessner, 2018), and thus employees’ task performance would not be improved. These results indicate that delivering high-quality feedback does not always lead to positive outcomes, and leaders should consider the congruence between leader delivered and employee expected feedback quality.

Moreover, learning goal orientation amplifies the positive indirect effects of congruence between expected feedback quality and delivered feedback quality on task performance and OCB through LMX, and exacerbates the negative effect of incongruence between expected feedback quality and delivered feedback quality on OCB *via* LMX. However, the results regarding the moderating role of learning goal orientation in the relationships between incongruence between expected feedback quality and delivered feedback quality and LMX and task performance partially differed from hypothesis 4 and hypothesis 5. We proposed that when incongruence is expected feedback quality exceeds delivered feedback quality, the negative effect of incongruence on LMX will be strengthened, while when incongruence is delivered feedback quality exceeds expected feedback quality, the negative effect of incongruence on LMX will be reduced for employees with high levels of learning goal orientation rather than low levels. However, the results indicate that whether expected feedback quality is greater than delivered feedback quality or *vice-versa*, the negative effect of incongruence between expected feedback quality and delivered feedback quality on LMX (*b_4_* = −1.85, *p* < 0.01 in Table 3) is exacerbated. A possible reason for such findings may be that high learning goal-oriented individual’s place importance on feedback quality and thus have a clear expectation of feedback quality. Oversupplying feedback quality (delivered feedback quality > expected feedback quality) is still seen by them as an additional workload, which is not beneficial for building a good exchange relationship with the leader. In addition, the results differ from hypothesis 5a in that learning goal orientation does not moderate the indirect relationship between incongruence between expected feedback quality and delivered feedback quality and task performance *via* LMX. This may be due to the targeted role of feedback quality on task performance.

In sum, those findings show that LMX can significantly mediate the relationship between congruence between expected feedback quality and delivered feedback quality and task performance, and learning goal orientation can strengthen those relationships. But LMX cannot play a mediating role in the relationship between incongruence between expected feedback quality and delivered feedback quality and task performance, even when learning goal orientation is high. On the one hand, this reflects the essential role of feedback quality on performance, and on the other hand, it reflects the difference between task performance and OCB.

### Theoretical implications

This study contributes to the literature in several ways. First, the majority of research on the effect of feedback on employee performance focused on their positive relationship. Although some scholars have broken down this prevalent argument and found that feedback is not always effective for performance (Murphy, 2020), few have empirically tested the reasons for this. Given the emphasis on the importance of employees’ feedback expectations in affecting employees’ reactions to feedback (Brett and Atwater, 2001), it is important to understand what employees expect from feedback and how such expectation affect their reactions after experiencing actual feedback quality from the leaders. By examining the influence of four situations (i.e., high expected feedback quality-high delivered feedback quality, low expected feedback quality-low delivered feedback quality, high expected feedback quality-low delivered feedback quality, and low expected feedback quality-high delivered feedback quality) on employee outcomes, our study not only provides a more precise picture of the relationship between feedback and employee outcomes and contributes to the feedback literature, but also provides evidence that employees perform better when the expected feedback quality is consistent with the delivered feedback quality. In brief, relative to improving the feedback quality itself, meeting employee feedback expectations has a greater impact on employee outcomes.

Second, this study is among the first to empirically investigate the implications of feedback on LMX and employee performance from the needs-supplies fit perspective. In the needs-supplies fit field, many scholars have matched many aspects of leaders and employees to study the influence of individual psychological needs and environmental supplies congruence on individual attitudes and behaviors. However, research on needs-supplies fit predominately focuses on general perceptions of need fulfillment (e.g., Marstand et al., 2017; Travaglianti et al., 2017; Klaic et al., 2018; Cao and Hamori, 2020; Yu and Davis, 2016), and only a few exceptions begin to explore the implications of the specific type from the perspective of employee needs. For example, Lambert et al. (2012) examined the effects of the fit between leader consideration and initiating structure needed and received on subordinates’ work-related attitudes. Rupprecht et al. (2016) investigated the influences of congruence between ideal and actual leader sensitivity on employees’ affects and counterproductive work behavior. Van Beurden et al. (2022) explored the effectiveness of HR practices for their job performance from the employee’s view. This study explores the implications of a new type of need fulfillment, such as employees’ feedback quality needs. That is, whether leaders’ delivered feedback quality fitting employees’ expected feedback quality affects employees’ work attitudes and work behaviors, enriching our understanding of need fulfillment.

Moreover, our results show that LMX mediates the relationship between congruence between expected feedback quality and delivered feedback quality and job performance, which has not been examined before to the best of our knowledge. On the one hand, this study enriched LMX research that LMX will improve as leaders give employees what they would like in their jobs (Marstand et al., 2017) by showing how leaders’ fulfillment of employee feedback quality needs affects LMX. On the other hand, prior studies examining the role of needs-supplies fit on employee outcomes have been mixed. For example, Martin et al. (2010) suggested that LMX quality is based on the mutual exchange of desired things about work, and for many parties, leaders just need to continue supplying more to employees regardless of how much employees would like. However, Marstand et al. (2017) argued that for some work-related needs, LMX was higher when leaders’ supplies and employees’ needs were consistent compared with inconsistence. In other words, the influence of needs-supplies fit on employee outcomes depends on the specific needs employees would like. Thus, this study provides new evidence in explaining the relationship between needs-supplies fit and employee outcomes from the feedback perspective.

Third, this study also extends the needs-supplies fit theory by identifying learning goal orientation as the contingency condition of the implications of congruence between expected feedback quality and delivered feedback quality. As scholars in the LMX field have constantly called for the need to examine potential moderators of the relationship between person-environment fit and outcomes (e.g., Marstand et al., 2017; Van Vianen, 2018). Therefore, such findings provide a more nuanced picture of how needs-supplies fit influence employee outcomes.

### Practical implications

The findings of this study have several valuable practical implications for leaders and employees. First, the results of the study show that employee task performance and OCB will be higher when leaders meet employees’ feedback quality needs at a high level rather than a low level. Thus, leaders and employees need to be more aware of the importance of *t* training programs focusing on learning about how to promote and meet employees’ feedback needs (Marstand et al., 2017). In addition, different from task performance, employee OCB will increase when leaders fulfill employees toward their level of need. Companies that value employee OCB are even more concerned with meeting employee feedback needs. Moreover, as also mentioned by Riggs and Porter (2017), leaders should make efforts to discuss with their employees about their behaviors (e.g., giving feedback) to better meet employees’ needs. What calls for special attention is that the feedback quality provided needs to be as high as possible based on satisfying employees.

Second, our findings highlight the important role of LMX in transmitting the influence of congruence between expected feedback quality and delivered feedback quality on job performance. Thus, LMX training should be included in leadership training programs. Earlier studies also confirmed the importance of training programs in the process of improving LMX, such as increasing management skills in social interaction and listening, so that leaders can empathize with their employees and understand their concerns and difficulties (Breevaart et al., 2015; Marstand et al., 2017). Moreover, employee feedback needs may change over time, and the interaction between the leader and the employee can reduce the psychological distance between the two parties, which can further benefit the more timely access to employee needs and finding a way to meet them sooner.

Finally, the research results show that high learning goal orientation accelerates the positive implications of congruence between expected feedback quality and delivered feedback quality. Although learning goal orientation is a relatively stable personality, it may gradually change over a longer period (Dahling and Ruppel, 2016). The organization can subtly shape the employees’ learning goal orientation by creating a team learning atmosphere (Dragoni, 2005). Moreover, in recruiting new employees, organizations could select employees with high learning goal orientation.

### Limitations and future research

This study has some limitations. First, the research design is cross-sectional in nature because of the short time duration (Bai et al., 2016; Li et al., 2021). Although our hypotheses were grounded in the existing theoretical rationale, causal relationships could not be drawn. For example, when LMX is high, leaders and employees can understand each other better, and the delivered feedback quality would be more matched with employees’ expectations. Consequently, experimental and longitudinal studies are suggested to examine our hypothesized relationships in future research.

Second, all variables were self-reported. However, to reduce the potential effect of common method variance (Podsakoff et al., 2003), we made the following efforts. We ensured the confidentiality and anonymity of the information provided by the participants. Besides, scales of variables were randomly presented in the survey to avoid participants from deducing the constructs’ cause-effect relationships. Moreover, we collect data at two points by 1 month apart. However, we still suggest that task performance and OCB are evaluated by direct leaders or others in future studies.

Third, other possible mechanisms and boundary conditions should be considered by further research. For example, according to social identity theory, when leaders’ delivered feedback quality meets employees’ expectations, employees may have a strong personal identification with leaders, and leader identification promotes employees to improve task performance and engage in more OCB (Wang and Howell, 2012). Moreover, future research could consider other goal orientations as boundary conditions. For example, performance goal orientation may mitigate the positive effects of congruence between expected feedback quality and delivered feedback quality because individuals with performance goal orientation believe that ability is a fixed attribute that is difficult to develop (VandeWalle, 2003), and pose little value to leaders’ feedback. Finally, in the analysis of the moderating process, we only discussed the moderating role of learning goal orientation in the relationship between congruence between expected feedback quality and delivered feedback quality and employee outcomes, ignoring the low vs. high congruence situations. This comparison can be achieved in the future by experimental methods.

## Data availability statement

The data analyzed in this study is subject to the following licenses/restrictions: the data that support the findings of this study are available from the corresponding author upon reasonable request. Requests to access these datasets should be directed to liuyy18@mail.nankai.edu.cn.

## Ethics statement

Ethical review and approval were not required for the study on human participants following the local legislation and institutional requirements. The participants provided their written informed consent to participate in this study.

## Author contributions

All authors have made a substantial, direct, and intellectual contribution to the work and approved it for publication.

## Funding

The authors disclosed receipt of the following financial support for the research, authorship, and/or publication of this article: This study was funded by the Digital Management and Behavioral Decision Art Laboratory, Shandong University of Finance and Economics, and Scientific Research and Innovation Projects for Tianjin Postgraduates (2020YJSB013), the National Natural Science Foundation Project (72072096) and the National Social Science Foundation of China (Grant number 19BJY037).

## Conflict of interest

The authors declare that the research was conducted in the absence of any commercial or financial relationships that could be construed as a potential conflict of interest.

## Publisher’s note

All claims expressed in this article are solely those of the authors and do not necessarily represent those of their affiliated organizations, or those of the publisher, the editors and the reviewers. Any product that may be evaluated in this article, or claim that may be made by its manufacturer, is not guaranteed or endorsed by the publisher.
